# Supplementary material for: MEKK-3 Acts Cooperatively with NSY-1 in SKN-1-Dependent Manner against Oxidative Stress and Aging in Caenorhabditis elegans
Source: Biology (Basel). 2022 Oct 18;11(10):1526. doi: 10.3390/biology11101526 (PMC9598901; doi:10.3390/biology11101526)
Supplement: Supplementary file 1 [file biology-11-01526-s001.zip › biology-1964724-supplementary.pdf]

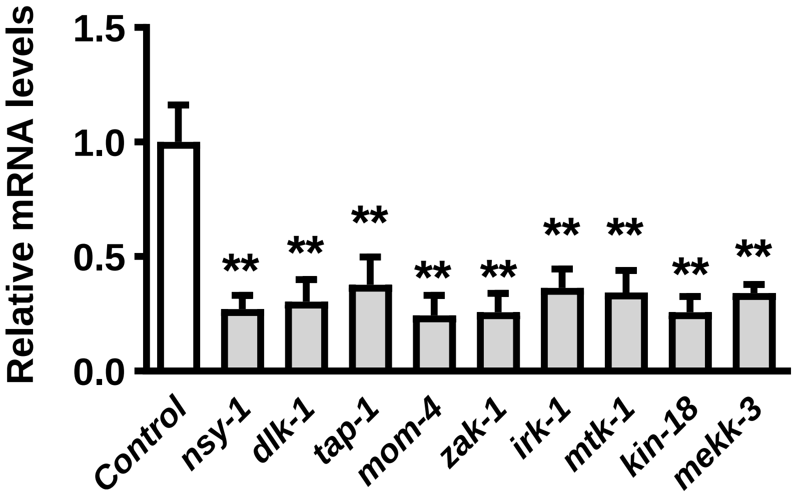

Figure S1. **Validation of RNAi silencing efficiency using quantitative PCR.** Quantitative PCR analyses were performed from *N2 Ex*[SKN-1::GFP] worms 24 h after fed with bacteria for each RNAi treatment (n = 3). The L4440 vector without insert in bacteria was used as a control. The fold-change in gene expression normalized to *act-1* and relative to the control sample was calculated using the $2^{-\Delta\Delta}$Ct method. Data are shown as the mean ± SEM. $^{**}p < 0.01$. All p values were calculated by ANOVA followed by Dunnett's multiple comparison tests.

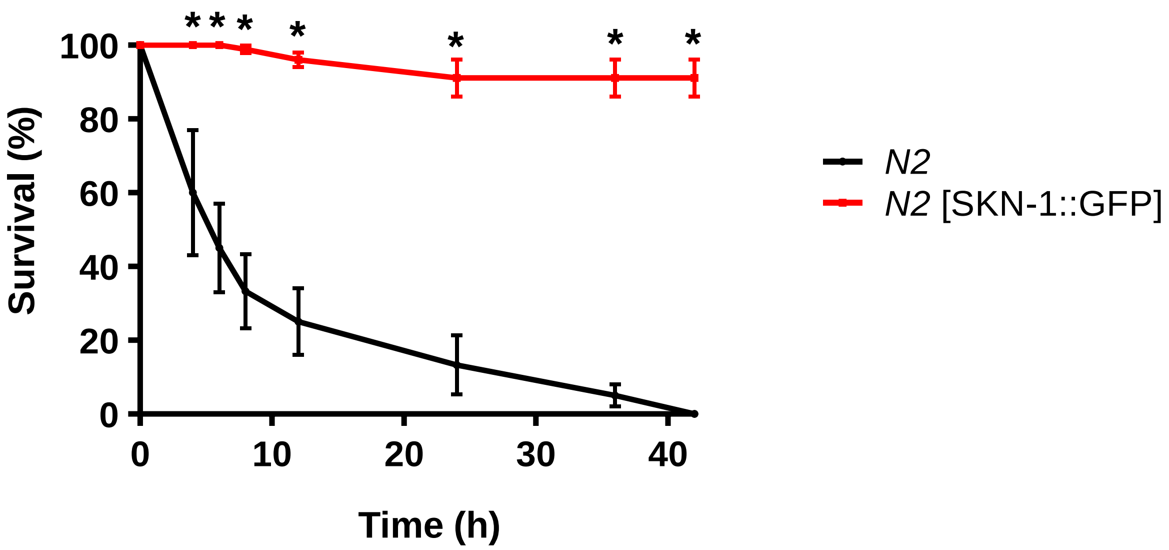

Figure S2. **Oxidative-stress-resistance assay of SKN-1 transgenic worms in 7.5 mM t-BOOH.** The survival rate was calculated at the indicated timepoints (h) after the exposure to 7.5 mM t-BOOH. Data are shown as the mean ± SEM. *$p < 0.01$. All p values were calculated by unpaired two-tailed Student's t tests.

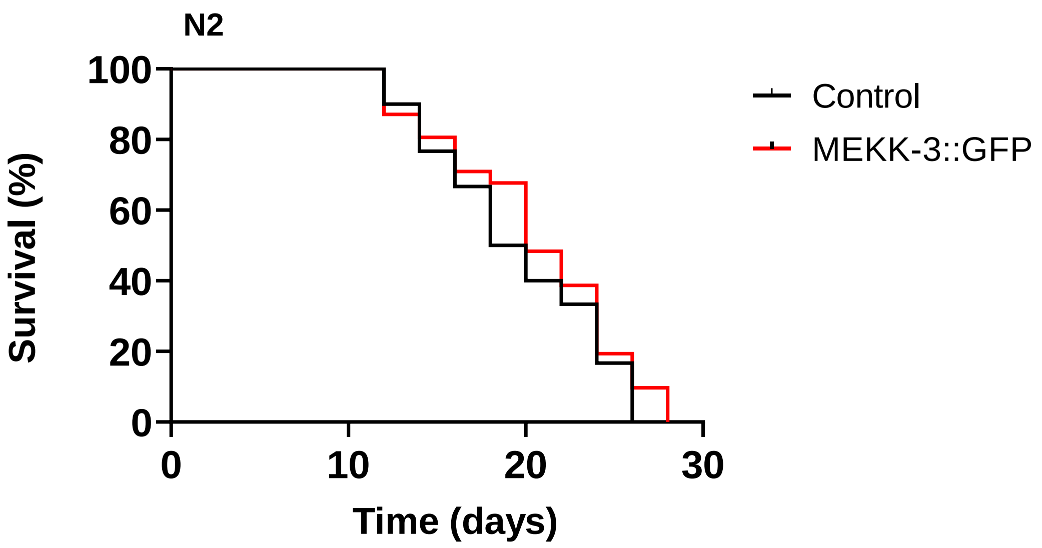

Figure S3. **Lifespan of MEKK-3 transgenic worms.** MEKK-3 transgenic young adult worms were transferred to plates with bacteria containing OP50. The percentage of live animals was plotted at the indicated timepoints (days). Data were combined from three experiments with n = 30 for each group (n = 90). The mean lifespan is shown in Table S2.

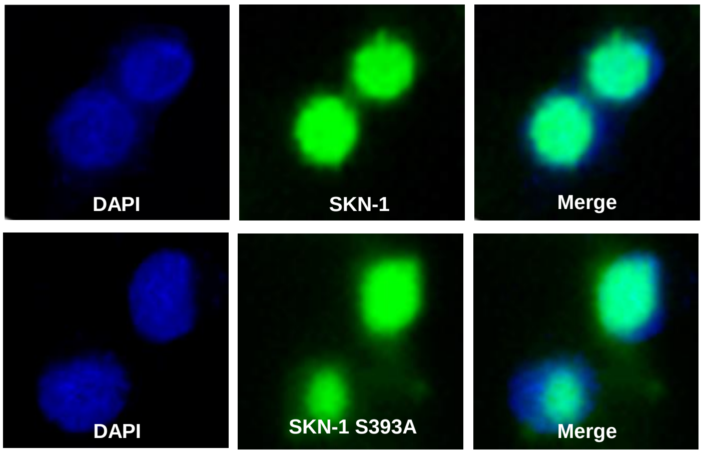

Figure S4. **Nuclear localization of SKN-1.** Upper images show SKN-1, and lower images show SKN-1 S393A in intestinal nuclei. Nuclei were counterstained with DAPI.

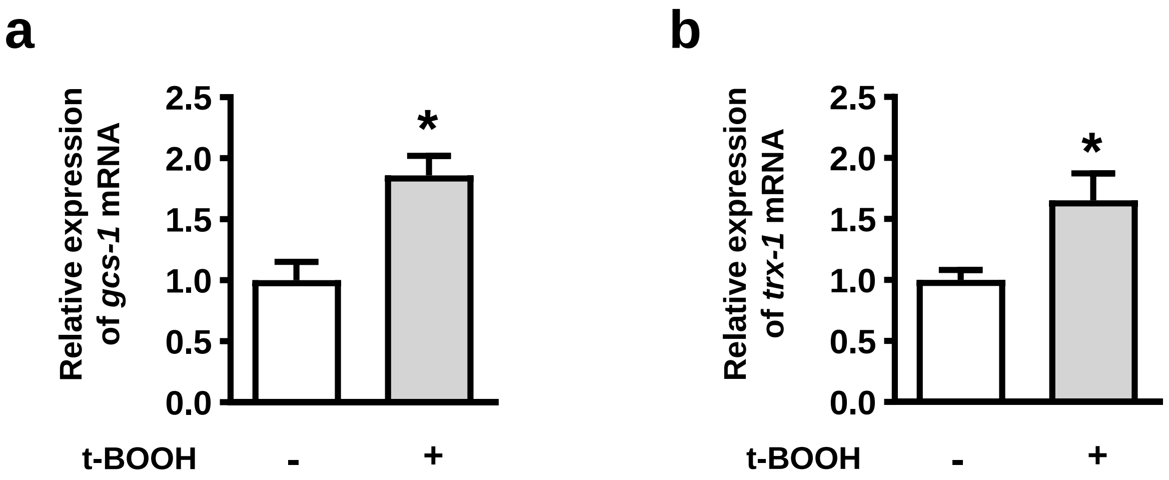

Figure S5. **mRNA expression of *gcs-1* and *trx-1* in oxidative stress.** Quantitative PCR analyses for *gcs-1* (**a**) and *trx-1* (**b**) were performed from *N2* worms 6 h after the exposure to 7.5 mM t-BOOH (n = 3). Data are shown as the mean ± SEM. *$p < 0.05$. All p values were calculated by unpaired two-tailed Student's t tests.

Table S1. The primer sequences for RT-qPCR analysis.

Gene	Gene ID	Direction	Primer sequence (5’-3’)
act-1	179535	Forward<br>Reverse	TCGGTATGGGACAGAAGGAC<br>CATCCCAGTTGGTGACGATA
mekk-3	172850	Forward<br>Reverse	GGAAGTTTTGGCGAGAGCAT<br>GCCTCGTCTAGCGTTTTCTG
nsy-1	24104671	Forward<br>Reverse	AGTTGGCTACGTGGATTGGA<br>CGACTTCGCATTGGAGACAG
gcs-1	174438	Forward<br>Reverse	GTTACAAGCCGAAGAGCAGG<br>AGCGATGAGACCTCCGTAAG
trx-1	181863	Forward<br>Reverse	TCTCTCACCAAGGAGCCTATTC<br>TTGCGTCTCCATTCTTGGTG
dlk-1	173128	Forward<br>Reverse	ACCACTCTCGACTTGGTCAC<br>CGACTTTTTCGTTGCATGGC
tap-1	181556	Forward<br>Reverse	CCTCGATCAATACCCTGCCA<br>CAAACAGGTGGGTTCGTTGT
mom-4	172842	Forward<br>Reverse	GTCCAGTCATTCGCCAAGTC<br>GCTGCTGGCCTAAAGTCATC
zak-1	175954	Forward<br>Reverse	CGCGAGAAAGCCCTTAAACA<br>CAAAAGTCGAGCGCCAGTTA
irk-1	180944	Forward<br>Reverse	TAGTGGAAGCCCATGTACGG<br>GAGGGCTGCGAGAATCAATG
mtk-1	172246	Forward<br>Reverse	CGAAGGATATGCGGCTTCAC<br>TTGGTCGGTGATTGGGATGA
kin-18	175862	Forward<br>Reverse	CTGGTGTTGGAACTATGCCG<br>TCGACGTGTCCAAAGGATCA

Table S2. Lifespan of *C. elegans* in this study.

Strain	Mean Lifespan ± SEM (days)	Median Lifespan (days)	*p* value	n
**Figure 3c**				
N2 Ex[SKN-1::GFP]; Control (RNAi)	24.45 ± 0.43	18	-	86
N2 Ex[SKN-1::GFP]; *mekk-3* (RNAi)	23.93 ± 0.46	18	0.6958[a]	81
N2 Ex[SKN-1::GFP]; *nsy-1* (RNAi)	23.60 ± 0.50	16	0.6970[a]	85
N2 Ex[SKN-1::GFP]; *mekk-3* (RNAi); *nsy-1* (RNAi)	18.87 ± 0.41	13	< .0001[a]<br>< .0001[b]<br>< .0001[c]	84
**Figure 3f**				
N2 Ex[SKN-1 S393A::GFP]; Control (RNAi)	27.07 ± 0.61	27	-	86
N2 Ex[SKN-1 S393A::GFP]; *mekk-3* (RNAi)	24.20 ± 0.53	24	0.0285[d]	86
N2 Ex[SKN-1 S393A::GFP]; *nsy-1* (RNAi)	23.87 ± 0.57	24	0.0260[d]	88
N2 Ex[SKN-1 S393A::GFP]; *mekk-3* (RNAi); *nsy-1* (RNAi)	19.80 ± 0.46	18	< .0001[d]<br>0.0004[e]<br>0.0009[f]	83
**Figure S3**				
N2	19.47 ± 0.50	19	-	86
N2 Ex[MEKK-3::GFP]	20.45 ± 0.54	20	0.3172[g]	87

SEM: standard error of the mean. n represents the total number of animals dying of old age versus those in the entire experiment (combined between trials, where appropriate). *p* values were calculated as follows: [a]*N2* Ex[SKN-1::GFP]; Control (RNAi), [b]*N2* Ex[SKN-1::GFP]; *mekk-3* (RNAi), [c]*N2* Ex[SKN-1::GFP]; *nsy-1* (RNAi), [d]*N2* Ex[SKN-1 S393A::GFP]; Control (RNAi), [e]*N2* Ex[SKN-1 S393A::GFP]; *mekk-3* (RNAi), [f]*N2* Ex[SKN-1 S393A::GFP]; *nsy-1* (RNAi), [g]*N2*. Statistical analyses for survival were conducted using Log-rank (Mantel-Cox) test.
